# Incidence and Risk Factors of Stump Complications Following Amputation in Patients With Diabetes: A Retrospective Analysis of the Nationwide Inpatient Sample

**DOI:** 10.1002/wjs.70357

**Published:** 2026-04-20

**Authors:** Yuanyuan Wang, Hong Ding, Xinlin Huang, Suyun Guo, Xiaohong Liu, Xiaofang Wang, Hao Xie, Ting Yang

**Affiliations:** ^1^ Department of Anesthesiology Beijing University of Chinese Medicine Shenzhen Hospital (Longgang) Shenzhen Guangdong China; ^2^ Department of Anesthesiology Nanfang Hospital Southern Medical University Guangzhou Guangdong China; ^3^ The Second School of Clinical Medicine Southern Medical University Guangzhou Guangdong China; ^4^ Nursing Department Beijing University of Chinese Medicine Shenzhen Hospital (Longgang) Shenzhen Guangdong China; ^5^ Division of Orthopaedic Surgery Department of Orthopaedics Nanfang Hospital Southern Medical University Guangzhou Guangdong China

**Keywords:** amputation, complication, diabetes, national inpatient sample

## Abstract

**Objective:**

To determine the incidence and risk factors associated with postoperative stump complications in diabetic patients who underwent amputation using a nationwide cohort study.

**Method:**

A retrospective cohort analysis was conducted using the Nationwide Inpatient Sample (NIS) database from 2010 to 2019. Patients were categorized into two groups based on the presence or absence of stump complications. Patient demographics (age, sex, and race), hospital characteristics (admission type, payer status, bed size, teaching status, location, and region), length of stay (LOS), total hospitalization charges, in‐hospital mortality, comorbidities, and perioperative complications were analyzed. Risk factors were identified using multivariate logistic regression analysis, incorporating patient demographics, hospitalization parameters, economic indicators, and comorbidities.

**Results:**

A total of 101,015 patients were included, of whom 6547 developed stump complications, yielding an overall incidence of 6.5%. Patients with stump complications had longer hospital stays (7 vs. 5 days, *p* < 0.0001) and higher total hospitalization charges ($52,248 vs. $40,226, *p* < 0.0001) than those without complications. Multivariable analysis showed that Black race, Hispanic ethnicity, Native American heritage, larger hospital bed size, greater comorbidity burden, weight loss, peripheral vascular disease, blood transfusion, hemorrhage/hematoma, wound dehiscence/non‐healing, and wound infection were independently associated with higher odds of stump complications.

**Conclusion:**

Several demographic, hospital‐level, and clinical factors were associated with postoperative stump complications in diabetic patients undergoing amputation. These findings may help improve perioperative risk stratification and identify patients who warrant closer postoperative monitoring.

## Highlights

- Significant Complication Rate: Stump complications occurred in 6.5% of 101,015 diabetic patients undergoing amputation during the study period, indicating a substantial clinical burden.
- Healthcare Resource Impact: Patients with stump complications demonstrated significantly extended hospital stays (7 vs. 5 days) and increased hospitalization costs ($52,248 vs. $40,226), emphasizing the substantial economic burden.
- Clinical Patterns: Peripheral vascular disease emerged as an important associated factor, while blood transfusion, hemorrhage/hematoma, wound dehiscence or non‐healing, and wound infection were also independently associated with higher odds of stump complications.
- Demographic Disparities: Multivariate analysis revealed higher risk among Black, Hispanic, and Native American patients, suggesting potential healthcare disparities requiring targeted interventions.
- Modifiable Risk Factors: Identification of adverse factors such as weight loss, peripheral vascular disease, transfusion, hemorrhage/hematoma, wound dehiscence/non‐healing, and wound infection may help guide perioperative risk assessment and postoperative monitoring.

## 1 Introduction

Diabetes mellitus (DM) is a major non‐communicable disease whose global prevalence continues to rise [1]. The number of adults with diabetes is projected to reach 643 million by 2030 and 783 million by 2045 [2]. As a common complication, diabetic foot ulcers (DFU) affect approximately 19%–34% of patients with diabetes [3], and about 20% of these patients ultimately require a lower‐extremity amputation (LEA) [4, 5], accounting for a major proportion of non‐traumatic amputations worldwide [6]. The consequences of amputation are severe, encompassing high mortality rates, significant socioeconomic burdens, and profound personal impacts such as declining physical function and diminished employment opportunities [7, 8]. These outcomes are frequently driven or exacerbated by a range of persistent, amputation‐related​ postoperative complications [9, 10].

Among the spectrum of postoperative issues, stump complications​ represent a significant challenge after LEA [9]. These complications primarily include stump infection, ulceration, necrosis, and osteomyelitis, which frequently necessitate additional surgical interventions or revision amputations [9, 11]. Their occurrence not only jeopardizes wound healing and prosthetic fitting but also substantially increases the risks of rehospitalization and further morbidity [12, 13]. Effectively managing these complications is therefore critical for improving functional recovery and overall outcomes in this vulnerable patient population.

While stump complications following amputation in diabetics can result in rehospitalization and increased healthcare costs, existing literature provides limited data regarding the risk factors, length of hospital stay (LOS), and mortality associated with these complications. This retrospective, multicenter study aimed to characterize the demographic and clinical features of diabetic patients who developed stump complications after amputation within the U.S. hospital network. Importantly, we sought to analyze the demographic data of diabetic patients experiencing stump complications post‐amputation and to elucidate the relationship between stump complications and mortality, LOS, and index admission characteristics. Overall, the present study sought to identify risk factors for stump complications and ultimately improve patient prognosis.

## 2 Methods

### 2.1 Data Source

The primary data source for this study was the Agency for Healthcare Research and Quality (AHRQ)‐sponsored Nationwide Inpatient Sample (NIS), administered through the Healthcare Cost and Utilization Project (HCUP). As the largest all‐payer inpatient database in the United States, the NIS comprises a 20% stratified sample of annual hospital discharges from over 1000 facilities. This study was exempt from Institutional Review Board (IRB) review as it involved the use of de‐identified data from the NIS, in accordance with U.S. federal regulations (45 CFR 46.104(d)(4)) and consistent with previous NIS‐based research [14, 15].

### 2.2 Data Collection

**FIGURE 1:**
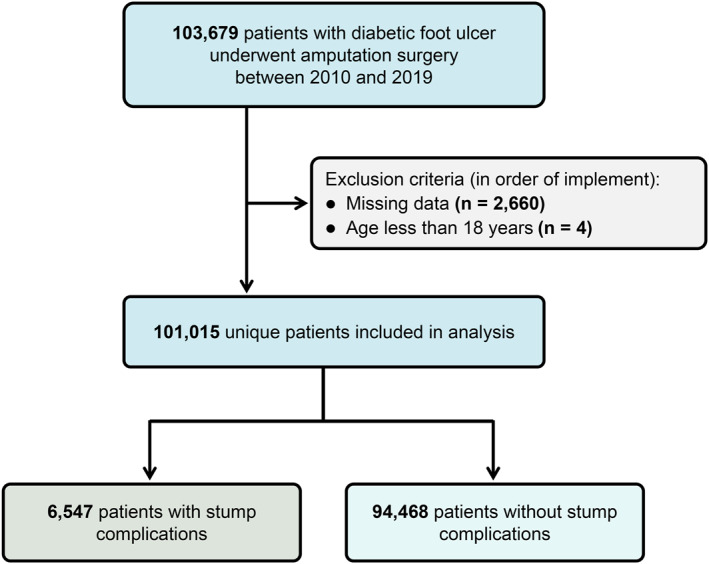
Flowchart of study cohort selection for the analysis of stump complications after amputation in patients with diabetes.

Data were obtained from the NIS database from 2010 to 2019. Patients with DFU were identified using International Classification of Diseases codes. In the International Classification of Diseases, Ninth Revision, Clinical Modification (ICD‐9‐CM), DFU was defined as 250.xx & 681.1x, 682.7, 707.14, 707.15, 730.07, and 730.17; in the International Classification of Diseases, Tenth Revision, Clinical Modification (ICD‐10‐CM), DFU was identified using E08.621, E09.621, E10.621, E11.621, E13.621, and Z86.31. Patients who underwent amputation were identified using the International Classification of Diseases, Ninth Revision, Procedure Coding System (ICD‐9‐PCS) codes 84.10–84.17 and the International Classification of Diseases, Tenth Revision, Procedure Coding System (ICD‐10‐PCS) codes 0Y6M0Z2‐0Y6M0Z8. Presence of at least one relevant diagnosis or procedure code within a discharge record was sufficient to define DFU or amputation, and each hospitalization was counted once. After excluding patients aged < 18 years or with missing key variables (Figure 1), the final study cohort was established.

### 2.3 Outcomes

The primary outcome was postoperative stump complications, defined using amputation stump‐specific ICD‐9‐CM and ICD‐10‐CM diagnosis codes. Stump complications were identified using ICD‐9‐CM codes 997.60, 997.62, and 997.69, and ICD‐10‐CM codes T87.43, T87.44, T87.81, T87.53, T87.54, T87.89, and T87.9. Postoperative stump complications were further classified as infection, dehiscence, necrosis, other specified, or unspecified stump complications (Table S1). Patients with codes corresponding to more than one stump complication subtype were classified as having multiple stump complications.

### 2.4 Data Analysis

Univariate analyses (*χ* ^2^ tests) were used to compare patient and hospital characteristics, comorbidities, and complications between patients with and without postoperative stump complications. Multivariable logistic regression models were constructed to identify independent risk factors (Table 1). To improve analytical sensitivity, additional multivariable logistic regression models were fitted for different types of stump complications, using covariates that were significant in the overall model, to assess subtype‐specific risk factors. Comorbidities were defined using the AHRQ comorbidity measures, and perioperative complications were identified based on ICD diagnosis codes. All analyses were performed using R software (version 4.3.2), with a two‐sided *p* value < 0.05 considered statistically significant.

**TABLE 1:** Variables used in binary logistic regression analysis.

Variables categories	Specific variables
Patient demographics	Age (≤ 64 years and ≥ 65 years), sex (male and female), race (White, Black, Hispanic, Asian or Pacific Islander, Native American and other)
Hospital characteristics	Type of admission (non‐elective, elective), bed size of hospital (small, medium, large), teaching status of hospital (nonteaching, teaching), location of hospital (rural, urban), type of insurance (medicare, medicaid, private insurance, self‐pay, no charge, other), region of hospital (northeast, Midwest or north central, south, west)
Comorbidities	AIDS, alcohol abuse, deficiency anemia, rheumatoid diseases, chronic blood loss anemia, congestive heart failure, chronic pulmonary disease, coagulopathy, depression, diabetes (uncomplicated), diabetes (with chronic complications), drug abuse, hypertension, hypothyroidism, liver disease, lymphoma, fluid and electrolyte disorders, metastatic cancer, neurological disorders, obesity, paralysis, peripheral vascular disorders, psychoses, pulmonary circulation disorders, renal failure, solid tumor without metastasis, peptic ulcer disease, valvular disease and weight loss

Abbreviation: AIDS, Acquired immunodeficiency syndrome.

## 3 Results

### 3.1 Incidence and Temporal Trends of Stump Complications

**FIGURE 2:**
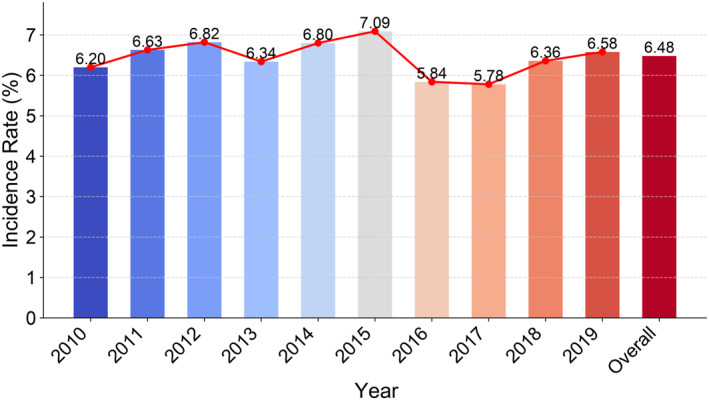
Annual and cumulative incidence of stump complications among patients with diabetes undergoing amputation from 2010 to 2019.

From 2010 to 2019, a total of 103,679 potentially eligible cases were extracted from the NIS database. After applying pre‐defined exclusion criteria, the final cohort consisted of 101,015 patients (Figure 1). Postoperative stump complications were identified in 6547 patients, resulting in an overall incidence rate of 6.5%. Among patients with stump complications, infection was the most frequent presentation (58.1%), followed by dehiscence (7.7%), multiple concurrent complications (3.9%), necrosis (2.0%), and other causes (28.3%) (Figure S1). The temporal trends in complication rates are depicted in Figure 2.

### 3.2 Patient Demographic Data

Among the 101,015 patients, the median age was 59 years in both groups, and age was not significantly associated with stump complications. Patients who developed stump complications were slightly more often male than those without complications (74.3% vs. 72.6%, *p* = 0.002). Racial distribution differed significantly, with a lower proportion of White patients and higher proportions of Black, Hispanic, and Native American patients in the complication group (*p* < 0.001). Medicare was the predominant primary payer in both groups, and patients with stump complications were marginally more likely to be covered by Medicare or Medicaid and less likely to have private insurance (*p* < 0.001) (Table 2).

**TABLE 2:** Univariable associations of patient and hospital characteristics with stump complications after amputation.

Characteristics	No stump complications	Stump complications	*p*
Total (*n* = count)	94,468	6547	
Total incidence (%)	6.50		
Age (median, years)	59 (51–68)	59 (52–68)	0.236
Age group (%)^a^			
18–44	12.00	11.47	0.485
45–64	53.69	53.92	
65–74	21.09	21.60	
≥ 75	13.22	13.01	
Sex (%)			
Male	72.56	74.34	0.002
Female	27.44	25.66	
Race (%)			
White	56.79	51.08	< 0.001
Black	19.39	22.32	
Hispanic	15.06	17.67	
Asian or Pacific Islander	1.18	1.22	
Native American	1.40	1.79	
Other	6.18	5.93	
Number of comorbidities (%)			
0	0.55	0.12	< 0.001
1	3.97	2.44	
2	11.88	9.59	
≥ 3	83.59	87.84	
LOS (median, d)	5 (3–9)	7 (4–11)	< 0.001
TOTCHG (median, $)	40,226 (22,027–73,488)	52,248 (29,005–95,312)	< 0.001
Type of insurance (%)			
Medicare	55.57	56.22	< 0.001
Medicaid	19.41	20.80	
Private insurance	17.82	16.31	
Self‐pay	4.42	4.35	
No charge	0.48	0.53	
Other	2.30	1.77	
Bed size of hospital (%)			
Small	17.46	15.76	< 0.001
Medium	28.22	27.54	
Large	54.33	56.70	
Elective admission (%)	14.43	16.73	< 0.001
Type of hospital (teaching %)	61.77	65.74	< 0.001
Location of hospital (urban, %)	91.27	94.12	< 0.001
Region of hospital (%)			
Northeast	18.98	20.19	< 0.001
Midwest or North Central	22.51	20.16	
South	37.84	39.50	
West	20.66	20.15	
Died (%)	0.94	0.66	0.02

Abbreviations: LOS, Length of stay; TOTCHG, Total charge.

^a^ Percentages are calculated based on the total number of patients within each column.

### 3.3 Hospital Characteristics

Following the development of stump complications, significant differences were observed across hospital bed size, hospital teaching status, and geographic region (*p* < 0.001) (Table 2).

### 3.4 Impact of Stump Complications on Hospital Stay and Mortality

A significant difference in preoperative comorbidity prevalence was observed between the two groups (*p* < 0.001). Patients with stump complications demonstrated a 1.4‐fold increase in hospital length of stay compared to those without complications. In‐hospital mortality also differed significantly between the two groups (*p* = 0.02) (Table 2).

### 3.5 Risk and Protective Factors Associated With Stump Complications

**FIGURE 3:**
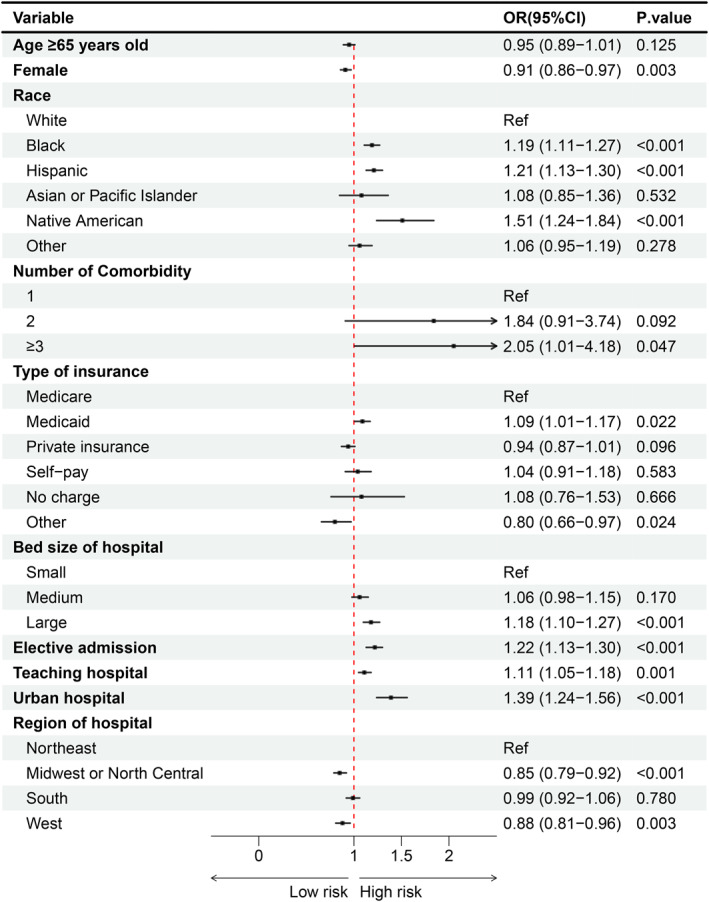
Associations between patient and hospital characteristics and stump complications.

Multivariable logistic regression identified Black race (odds ratio [OR] 1.19; 95% confidence interval [CI] 1.11–1.27), Hispanic ethnicity (OR 1.21; 95% CI 1.13–1.30), Native American race (OR 1.51; 95% CI 1.24–1.84), having ≥ 3 comorbidities (OR 2.05; 95% CI 1.01–4.18), Medicaid insurance (OR 1.09; 95% CI 1.01–1.17), large hospital bed size (OR 1.18; 95% CI 1.10–1.27), elective admission (OR 1.22; 95% CI 1.13–1.30), teaching hospital status (OR 1.11; 95% CI 1.05–1.18), and urban hospital location (OR 1.39; 95% CI 1.24–1.56) as independent risk factors for stump complications (all *p* < 0.05). In contrast, female sex (OR 0.91; 95% CI 0.86–0.97), “other” insurance (OR 0.80; 95% CI 0.66–0.97), and hospital location in the Midwest (OR 0.85; 95% CI 0.79–0.92) or West (OR 0.88; 95% CI 0.81–0.96) were associated with lower odds of stump complications (all *p* < 0.05) (Figure 3).

### 3.6 Preoperative Comorbidities

**FIGURE 4:**
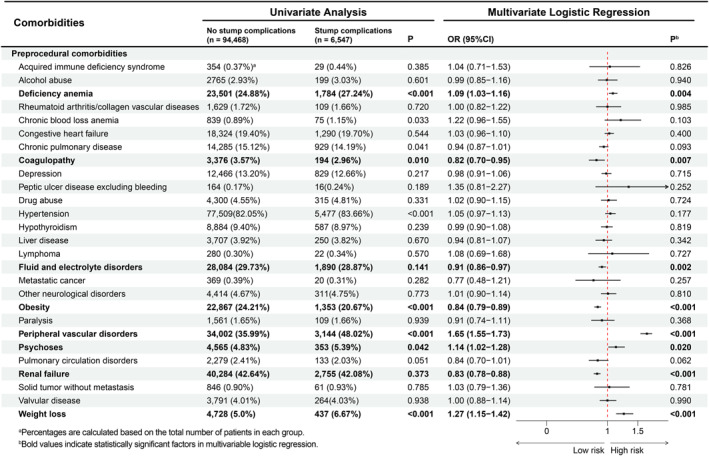
Associations between pre‐existing comorbidities and the development of stump complications.

As shown in Figure 4, univariate analysis demonstrated significant between‐group differences in several preprocedural comorbidities: patients with stump complications more frequently had deficiency anemia, chronic pulmonary disease, coagulopathy, hypertension, peripheral vascular disorders, psychoses, and weight loss, whereas obesity was less common in this group (all *p* < 0.05). In multivariable logistic regression, deficiency anemia (OR 1.09; 95% CI 1.03–1.16), peripheral vascular disorders (OR 1.65; 95% CI 1.55–1.73), psychoses (OR 1.14; 95% CI 1.02–1.28), and weight loss (OR 1.27; 95% CI 1.15–1.42) were associated with higher odds of stump complications, whereas coagulopathy (OR 0.82; 95% CI 0.70–0.95), fluid and electrolyte disorders (OR 0.91; 95% CI 0.86–0.97), obesity (OR 0.84; 95% CI 0.79–0.89), and renal failure (OR 0.83; 95% CI 0.78–0.88) were associated with lower odds (Figure 4).

### 3.7 Postoperative Complications

**FIGURE 5:**
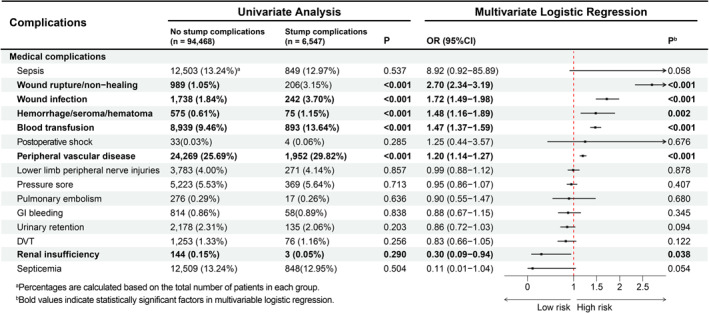
Associations between perioperative complications and the risk of stump complications.

As shown in Figure 5, univariate analysis demonstrated that patients with stump complications more frequently developed wound rupture/non‐healing, wound infection, hemorrhage/seroma/hematoma, required blood transfusion, and had peripheral vascular disease compared with those without stump complications (all *p* < 0.001). In multivariable logistic regression, wound rupture/non‐healing (OR 2.70; 95% CI 2.34–3.19), wound infection (OR 1.72; 95% CI 1.49–1.98), hemorrhage/seroma/hematoma (OR 1.48; 95% CI 1.16–1.89), blood transfusion (OR 1.47; 95% CI 1.37–1.59), and peripheral vascular disease (OR 1.20; 95% CI 1.14–1.27) were associated with higher odds of stump complications, whereas renal insufficiency (OR 0.30; 95% CI 0.09–0.94) was associated with lower odds (Figure 5).

### 3.8 Stratified Sensitivity Analysis of Subtype‐Specific Associations

In the stratified sensitivity analysis, most associations remained directionally consistent with the overall model, but several predictors exhibited notable effect modification. Deficiency anemia, a risk factor in the primary analysis, showed a reversed direction for dehiscence and necrosis, while still indicating increased risk for infection. Renal failure, previously protective, likewise shifted toward a risk‐increasing pattern in dehiscence. Several predictors significant in the overall model—such as psychoses, fluid/electrolyte disorders, and certain perioperative complications—lost significance in subtype‐specific models, indicating outcome‐specific variability. In contrast, weight loss consistently remained an adverse factor across all subtypes, supporting the robustness of this association (Figure S2).

## 4 Discussion

This study provides a comprehensive analysis of stump complications following amputation in patients with diabetes, focusing on associated health factors and economic impacts. Utilizing a nationwide inpatient database, the incidence of postoperative stump complications was examined from 2010 to 2019, revealing a range of 5.8%–7.1%. A peak incidence was observed in 2015, followed by a decrease to 5.8%–6.6% after 2016. This observed decrease may be attributed to the transition from ICD‐9‐CM to ICD‐10‐CM coding after October 2015, potentially leading to underreporting of postoperative complications in some institutions.

In multivariable analyses, Black, Hispanic, and Native American patients were associated with a higher occurrence of postoperative stump complications. This finding should be interpreted cautiously, because race and ethnicity are widely regarded as social constructs that more often capture differences in socioeconomic conditions, access to care, insurance coverage, and structural inequities rather than inherent biological risk [16]. This interpretation is consistent with prior work in diabetic foot disease and LEA, where delayed presentation, limited availability of limb‐salvage procedures (e.g., revascularization), and disparities in perioperative care have been shown to contribute to worse outcomes among minoritized populations, mechanisms that may partially account for the stump complication differences observed in our cohort [17]. It should also be noted that, within the NIS framework, race and ethnicity are coded as administrative variables, and the lack of granular information on social determinants and care pathways restricts causal inference and likely leaves residual confounding [18]. Future studies incorporating richer social‐determinant and care‐process data are needed to further elucidate the pathways underlying these associations.

Hospital‐level characteristics were also significantly associated with stump complications. Higher recorded odds were observed among patients treated at large, teaching, and urban hospitals, as well as among those admitted electively, compared with patients treated at small, non‐teaching, rural hospitals or admitted emergently. These findings likely reflect the referral of more complex and high‐risk cases to large academic and urban centers, along with more comprehensive documentation and coding of secondary diagnoses and complications, rather than true differences in quality of care. Consistent with prior studies, teaching hospitals tend to manage patients with greater clinical complexity and exhibit more intensive coding practices than community hospitals [19].

In our cohort, women had slightly lower rates of stump complications than men, and female sex was independently associated with reduced odds of stump complications. This gradient is consistent with prior cohort studies and meta‐analyses showing that men with diabetes have a higher risk of LEA than women [20, 21]. Sex‐related differences in vascular disease, neuropathy, and health behaviors have been proposed as potential mechanisms for these disparities [22]. Together, these data suggest that female sex may confer a modest protective effect on post‐amputation stump outcomes.

Weight loss is a recognized clinical marker of disease‐related malnutrition and frailty in patients with chronic illness [23]. Clinical studies have shown that low BMI and weight loss are associated with higher risks of LEA and adverse postoperative outcomes in patients with diabetes and vascular disease [6]. In our cohort, the comorbidity of weight loss consistently remained an adverse factor across all stump complication subtypes, supporting the notion that catabolic nutritional states and muscle wasting may impair wound healing and stump integrity. These findings underscore the importance of early nutritional assessment and targeted intervention in this high‐risk population.

Peripheral artery disease (PAD) develops from atherosclerosis in lower limb arteries, causing vascular stenosis or obstruction. Chronic limb‐threatening ischemia, the most advanced PAD stage, increases amputation and cardiovascular death risks. Diabetes exacerbates PAD through higher prevalence, accelerated progression, and worse severity. The combination of diabetes and PAD greatly elevates the likelihood of adverse outcomes, especially LEA [24]. Consistent with this pathophysiologic framework, pre‐existing peripheral vascular disease in our diabetic cohort emerged as a strong independent risk factor for postoperative stump complications.

Local wound failure and bleeding‐related events were among the strongest predictors of stump complications. Specifically, wound dehiscence or non‐healing and surgical‐site infection indicate failure of primary closure and ongoing contamination, which can prolong inflammation, delay epithelialization, and often necessitate repeated debridement or revision amputation—findings that are consistent with prior reports identifying dehiscence and stump infection as leading causes of adverse residual‐limb outcomes after major amputation [25]. Postoperative hemorrhage, seroma/hematoma, and the need for transfusion can increase tissue pressure, compromise microcirculatory perfusion, create a favorable medium for bacterial growth, and exacerbate tissue hypoxia, all of which impair stump healing and increase morbidity and length of stay [26]. These findings highlight meticulous hemostasis and early recognition and treatment of wound complications as central targets for reducing residual‐limb problems in patients undergoing diabetes‐related amputation.

In our cohort, age was not significantly associated with postoperative stump complications, either across prespecified age groups (*χ* ^2^ *p* = 0.485) or in multivariable models. Similar attenuation of age effects after adjustment for comorbidities and operative factors has been reported in amputation and vascular surgery studies [27, 28], suggesting that age‐related risk is largely mediated through these more proximal clinical factors. Thus, our findings indicate that comorbidity burden and disease severity, rather than chronological age alone, are more strongly associated with stump complications.

A similarly counterintuitive pattern was observed for renal failure, which was associated with slightly lower odds of stump complications in multivariable models. Patients with renal failure have high competing risks of early death or readmission for other causes, shortening the window in which stump complications can be observed and coded in administrative data [29, 30]. Thus, the inverse association is likely driven by selection and residual confounding rather than a true protective effect, a view supported by subtype‐specific analyses (Figure S2), in which renal failure was linked to higher odds of dehiscence but lower odds of infection.

Several other predictors also showed inverse or attenuated associations in the overall model. Coagulopathy and fluid and electrolyte disorders were inversely associated with overall stump complications; however, subtype analyses indicated that their effects were confined to stump infection or became non‐significant across complications, so these results should not be interpreted as true protective effects. By contrast, obesity was consistently associated with lower odds of stump complications, in keeping with the “obesity paradox,” whereby higher body weight in high‐risk surgical patients may reflect better nutritional reserve and compensatory capacity [31].

Given the large sample size, we acknowledge that some statistically significant associations may have limited clinical relevance. Several predictors exhibited only modest effect sizes (ORs around 1.1), particularly certain payer, hospital, and comorbidity variables, and stratified analyses indicated that these smaller effects often attenuated or varied across stump‐complication subtypes. These findings should therefore be interpreted with caution. By contrast, predictors with larger effect sizes—such as weight loss and key perioperative wound events (dehiscence/non‐healing, infection, hemorrhage/hematoma, transfusion)—showed stronger and more consistent associations with stump complications, suggesting that these factors may be more relevant for clinical risk stratification and closer postoperative surveillance.

This study has several limitations that should be acknowledged. First, the retrospective observational design is susceptible to selection bias and unmeasured confounding, inherent limitations in non‐randomized studies. Second, while the NIS database offers a large sample size, its reliance on administrative coding may introduce inaccuracies in diagnostic and procedural classifications, as well as occasional missing data. Third, the NIS analyzes hospitalization events rather than individual patients, precluding the identification of readmissions within the study period and potentially leading to duplicate patient entries. Fourth, the exclusion of outpatient records limits the analysis to inpatient amputations, potentially missing minor amputations performed in ambulatory settings. Finally, the inclusion of in‐hospital mortality cases among diabetic patients undergoing LEAs may have influenced race/ethnicity‐stratified amputation rate estimates. While mortality outcomes were outside the scope of this study, they represent a critical area for future research.

## 5 Conclusion

In this nationwide cohort of diabetic patients undergoing amputation, the incidence of stump complications was 6.5%. Several sociodemographic, health system, and clinical factors were associated with higher odds of stump complications. These included Black race, Hispanic ethnicity, Native American heritage, treatment in large hospitals, and clinical factors such as higher overall comorbidity burden, peripheral vascular disease, blood transfusion, hemorrhage/hematoma, non‐healing wounds, and wound infection. These findings highlight the clinical burden of stump complications and may assist clinicians in recognizing patients at increased risk, refining perioperative risk stratification, and improving postoperative surveillance.

## Author Contributions

**Yuanyuan Wang:** software, methodology, writing – original draft, project administration, investigation, data curation. **Hong Ding:** data curation, formal analysis, writing – review and editing. **Xinlin Huang:** software, data curation, formal analysis, writing – original draft, methodology, visualization. **Suyun Guo:** software, methodology, data curation. **Xiaohong Liu:** validation, software, visualization. **Xiaofang Wang:** writing – original draft, data curation, software. **Hao Xie:** supervision, validation, writing – review and editing. **Ting Yang:** writing – review and editing, project administration, supervision, resources, conceptualization.

## Funding

This study was supported by the Clinical Research Program of Nanfang Hospital, Southern Medical University (2022) (Grant No. 2021CR027), and the Basic and Applied Basic Research Program of Guangzhou Science and Technology Project (Grant No. 2023A04J2333).

## Conflicts of Interest

The authors declare no conflicts of interest.

## Supporting information


**Figure S1:** Distribution of different types of stump complications.

**Figure S2:** Stratified sensitivity analysis of predictors across different types of stump complications.

**Table S1:** ICD codes for diseases and procedures used in cohort definition.

## Data Availability

The data that support the findings of this study are available from the corresponding author upon reasonable request.
