# Supplementary figures and images for: Incidence and Risk Factors of Stump Complications Following Amputation in Patients With Diabetes: A Retrospective Analysis of the Nationwide Inpatient Sample
Source: World J Surg. 2026 Apr 20;50(6):1714–23. doi: 10.1002/wjs.70357 (PMC13242060; doi:10.1002/wjs.70357)

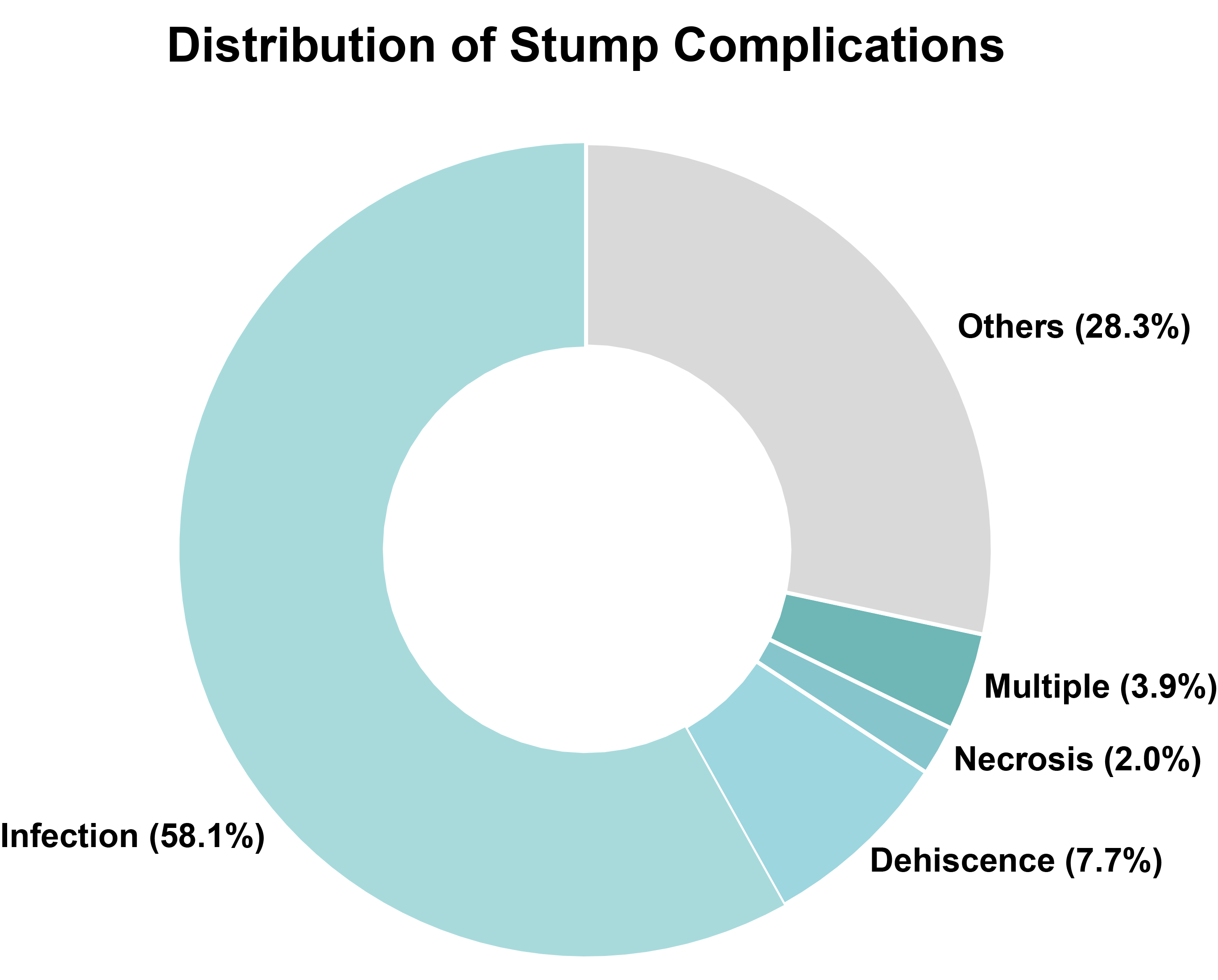

Supplement: Supplementary file 1 — Figure S1: Distribution of different types of stump complications. [file WJS-50-1714-s002.tif]

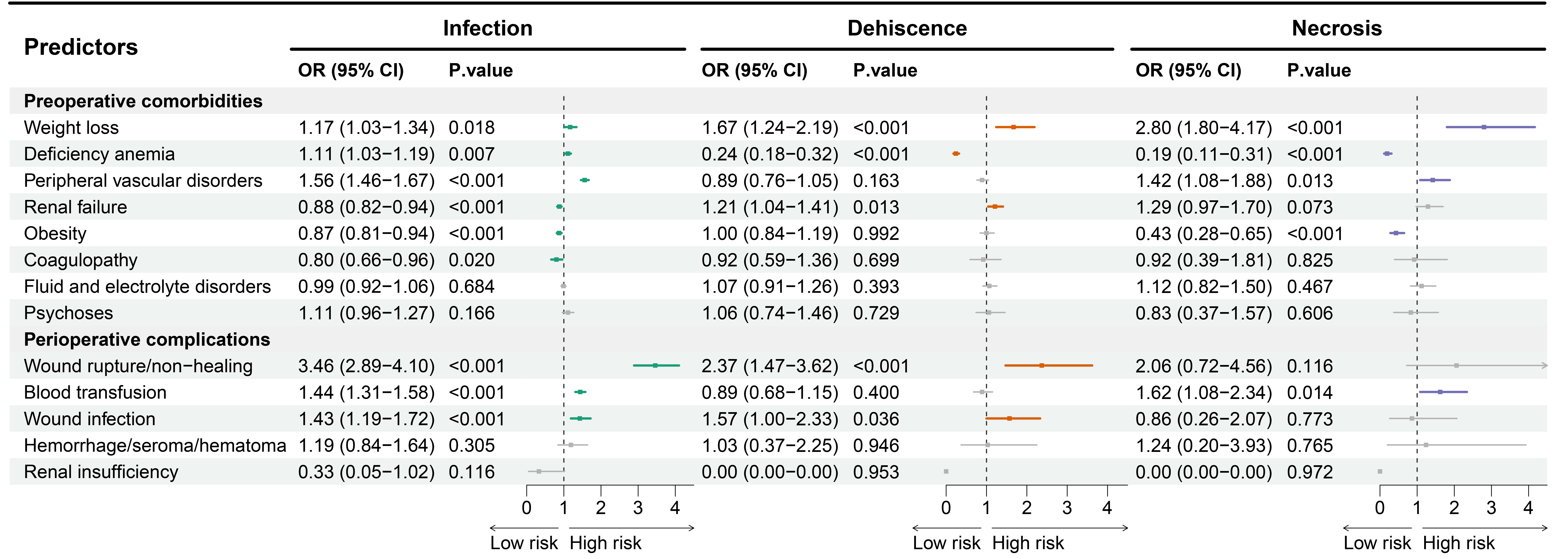

Supplement: Supplementary file 2 — Figure S2: Stratified sensitivity analysis of predictors across different types of stump complications. [file WJS-50-1714-s003.tif]
