# Supplementary material for: Incidence and Risk Factors of Stump Complications Following Amputation in Patients With Diabetes: A Retrospective Analysis of the Nationwide Inpatient Sample
Source: World J Surg. 2026 Apr 20;50(6):1714–23. doi: 10.1002/wjs.70357 (PMC13242060; doi:10.1002/wjs.70357)
Supplement: Supplementary file 3 — Table S1: ICD codes for diseases and procedures used in cohort definition. [file WJS-50-1714-s001.docx]

**Table S1. ICD codes for diseases and procedures used in cohort definition**

| **Diseases / Procedures** | | **ICD-9 codes** | **ICD-10 codes** |
| --- | --- | --- | --- |
| **Diabetic foot ulcer** | | 250.xx & 681.1x, 682.7, 707.14, 707.15, 730.07, 730.17 | E08.621, E09.621, E10.621, E11.621, E13.621, Z86.31 |
| **Amputation surgery** | | 84.10, 84.11, 84.12, 84.13, 84.14, 84.15, 84.16, 84.17 | 0Y6M0Z2, 0Y6M0Z3, 0Y6M0Z4, 0Y6M0Z5, 0Y6M0Z6, 0Y6M0Z7, 0Y6M0Z8 |
| **Stump complications following amputation** | **Infection of amputation stump** | 997.62 | T87.43, T87.44 |
|  | **Dehiscence of amputation stump** | NA | T87.81 |
|  | **Necrosis of amputation stump** | NA | T87.53, T87.54 |
|  | **Other complications of amputation stump** | 997.69 | T87.89 |
|  | **Unspecified complications of amputation stump** | 997.60 | T87.9 |
